# Initial evaluation of a four‐dimensional computed tomography system, using a programmable motor

**DOI:** 10.1120/jacmp.v7i4.2301

**Published:** 2006-11-28

**Authors:** Luc Simon, Philippe Giraud, Vincent Servois, Jean‐Claude Rosenwald

**Affiliations:** ^1^ Institut Curie Département de Radiothérapie Paris France; ^2^ Institut Curie Département d'Imagerie Médicale Paris France

**Keywords:** 4D‐CT, programmable phantom, quality control, organ motion, tomography

## Abstract

A dynamic lung tumor phantom was used to investigate the geometric reconstruction accuracy of a commercial four‐dimensional computed tomography (4D‐CT) system. A ball filled with resin, embedded in a cork cube, was placed on a moving platform. Various realistic antero‐posterior (AP) motions were programmed to reproduce the respiratory motion of a lung tumor. Several three‐dimensional (3D) CT and 4D‐CT images of this moving object were acquired and compared using different acquisition parameters. Apparent volume and diameter of the ball were measured and compared to the real values. The position of two points (the AP limits of the ball) during the motion in the coordinate system of the CT scanner were also compared with the expected values. Volume error was shown to increase with object speed. However, although the volume error was associated with intraslice artifacts, it did not reflect large interslice inconstancies in object position and should not be used as an indicator of image accuracy. The 3D‐CT gave a random position of the tumor along the phantom excursion; accuracy in the assessment of position by 4D‐CT ranged from 0.4 mm to 2.6 mm during extreme phases of breathing. We used average projection (AVE) and maximum intensity projection (MIP) algorithms available on the commercial software to create internal target volumes (ITVs) by merging gross tumor volume (GTV) images at various respiratory phases. The ITVs were compared to a theoretical value computed from the programmed ball excursion. The ITVs created from the MIP algorithm were closer to the theoretical value (within 12%) than were those created from the AVE algorithm (within 40%).

PACS numbers: 87.53.Xd, 87.56.Mp, 87.57.Ce, 87.59.Fm

## I. INTRODUCTION

The importance of respiratory motion in radiotherapy of tumors located in the chest has been reported in numerous publications.^(^ ^1^ ^–^ ^4^ ^)^ It is now commonly admitted that breathing‐adapted radiotherapy should improve local control by increasing the accuracy of targeting—for example, for non‐small‐cell lung cancer.^(5)^ If no significant improvements in survival have yet been reported using these techniques, several studies have highlighted better dose conformity and a reduction in dose to organs at risk (OARs).^(^ ^6^ ^–^ ^10^ ^)^

Generally, these new techniques that manage the patient's respiratory motion during treatment are separated into two groups: breath‐hold techniques^(^ ^6^ ^,^ ^11^ ^)^ and gating techniques.^(12)^ In the breath‐hold technique, the patient is imaged and treated during a monitored breath‐hold. This approach prevents motion of the tumor and the OARs. Moreover, if breath‐hold is achieved at deep inspiration, dosimetric benefits have also been observed for lung.^(6)^ In the gating technique, the patient is treated at a particular phase of free breathing, generally by placing an external marker on the patient's chest and synchronizing beam delivery with the motion of the marker.^(^ ^12^ ^–^ ^14^ ^)^ For this group of patients, the treatment plan should be based on a breathing‐adapted imaging technique. Four‐dimensional computed tomography (4D‐CT) is such a technique^(^ ^15^ ^–^ ^19^ ^)^ and the focus of the present work.

With the use of breathing‐adapted techniques, dose escalation is envisaged because of the reduction in target volume and, consequently, in toxicity to the OARs.^(^ ^5^ ^,^ ^20^ ^)^ However, to reach this goal safely, radiation oncologists must first be able to precisely control treatment accuracy with respect to tumor location. Indeed, in the case of therapy with a reduced target volume, small geometric uncertainties could lead to large dosimetric errors—underdosage of tumor and overdosage of OARs.

These concerns led us to implement specific quality control procedures. By performing a cross‐comparison between spirometer and 4D‐CT, we demonstrated how lung volume could be obtained accurately from either technique.^(21)^ On the other hand, Rietzel et al. used a dynamic phantom animated with a sinusoidal motion to study the influence of superior–inferior (SI) motion on 4D‐CT images. For a regular motion along that direction, they found that, with 4D‐CT, object position can be determined with an accuracy equal to about one slice thickness (2.5 mm).^(22)^ Motions along the SI direction lead to spiral‐shaped blurring.^(23)^ Other studies on CT artifacts induced by SI motion exist, but practically no studies on AP motion artifacts can be found.

The aim of the present work was to study the consequences on CT images of AP motion. We considered a variation pattern that was not sinusoidal, but that was adjusted according to typical clinical data: inspiration is shorter than expiration.^(24)^

Motions along the AP direction are generally considered to be smaller than those along the SI direction. However, Ross et al. measured AP tumor motion that could reach 22 mm.^(25)^ Giraud et al. also observed, for one patient, an AP motion of 37 mm during deep breathing.^(26)^ In practice, actual tumor motion is a combination of AP and SI motions. However, in the context of a phantom study, we think that AP and SI motions should be studied separately to permit a better understanding of the origin of each type of artifact.

A further reason for investigating AP movement comes from the fact that the marker used as a surrogate for breathing [Real‐time Position Manager (RPM: Varian Medical Systems, Palo Alto, CA)] moves principally along the AP direction. The corresponding excursion therefore provides a value that can be considered the maximum excursion of an internal lung tumor attached to the chest wall.

The AP motion leads to artifacts that are different in nature from artifacts attributable to SI motion. As will be discussed later, AP motion does not induce spiral‐shaped artifacts, but rather intraslice vertical blurring and interslice inconsistencies. Intraslice blurring is induced by the motion of the object during the acquisition of one slice (one tube rotation). Interslice inconsistencies reflect the fact that an object can have different positions during the acquisition of two consecutive slices. These differences are not clearly recognized in the literature. We therefore developed a dynamic lung tumor phantom with a simple and known geometry.

A ball filled with resin was embedded in a cork cube and placed on the platform of a programmable motor that can create complex AP motions. Realistic breathing motions were programmed. Several CT acquisitions of the phantom were obtained with and without motion. Artifacts were estimated by measuring both the volume and the position of the ball in the CT referential. These measurements allowed us to understand and to quantify the effects of AP motion on images. We assessed the benefits of 4D‐CT as compared with standard 3D‐CT. In addition, we used two different algorithms to merge images at varying phases of the breathing cycle. These studies allowed us to evaluate the ability of 4D‐CT to create an individualized internal target volume (ITV) encompassing the moving tumor during gated or non‐gated treatment.

## II. MATERIALS AND METHODS

### A. 4D‐CT principle

Using 4D‐CT, a full $N_{s}$ series (e.g., 10) 3D‐CT images can be built, each corresponding to a different phase of a patient's breathing cycle. Four‐dimensional CT has been fully described in the literature.^(^ ^22^ ^,^ ^27^ ^)^ It is performed using a specific acquisition mode of CT (cine mode) available on the LightSpeed QX/i CT (GE Healthcare, Waukesha, WI) and a software program to sort images (Advantage4D, v1.4, GE Healthcare), according to the position of an external marker that follows the motion of the chest wall.

#### A.1 External marker

We currently use the RPM system for both imaging and gated treatment. The RPM system has been described in the literature.^(^ ^12^ ^,^ ^13^ ^)^ It is based on an external reflecting tape stuck on a small, lightweight cube and followed by an infrared camera. The cube is usually positioned on the patient's chest and is considered a surrogate of tumor motion. The RPM system acquires 1D motion $\left(25\;\text{samples}•s^{-1}\right)$ as a file that contains mainly a time stamp, marker amplitude, and corresponding phase. Linear accelerators can be synchronized to this system to deliver a gated treatment. The same system is used to sort 4D images.

#### A.2 Definition of phase and phase tolerance

**Figure 1:**
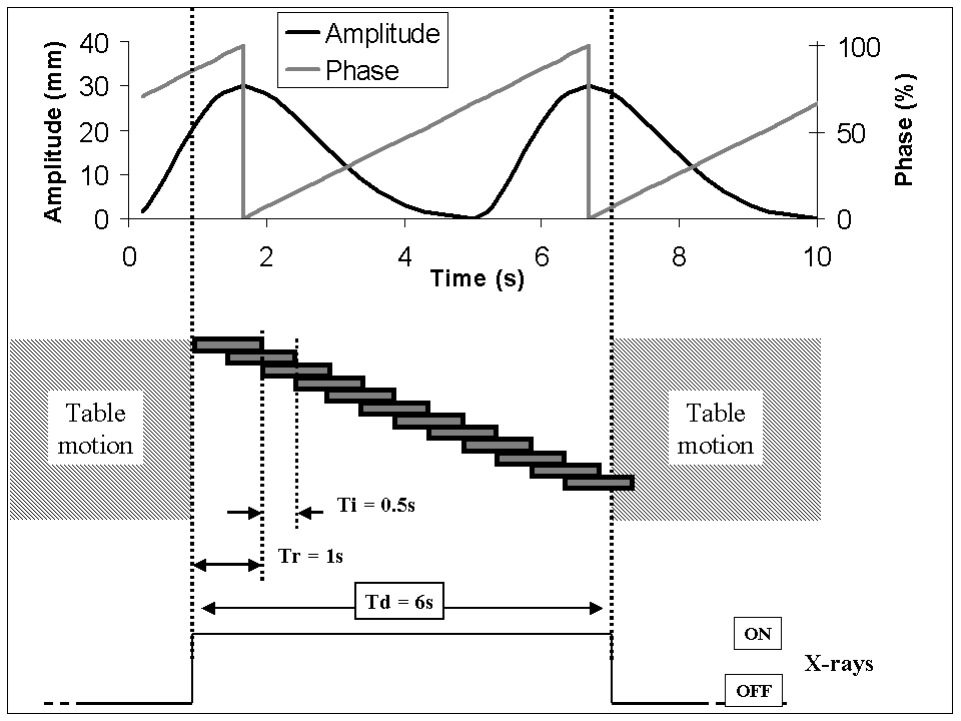
Principle of acquisition of one slice using four‐dimensional computed tomography (4D‐CT); $T_{d}$, $T_{r}$, and $T_{i}$ are set, for example, to 6 s, 1 s, and 0.5 s respectively. At the top of the figure, amplitude and phase of a typical breathing signal acquired by Real‐time Position Manager (Varian Medical Systems, Palo Alto, CA) are plotted against time. At the bottom of the figure, each solid rectangle represents one image acquisition. In this configuration, 11 images can be fully reconstructed during a cine duration $\left(T_{i}\right)$. After this sequence, the table moves to the next position. A rectangle can represent more than one image in the case of multislice CT. $T_{d}$ is chosen to be greater than one breathing cycle

“Phase” is defined as 0% at the beginning of a breathing cycle—that is, at end inspiration (EI)—and 100% at the next EI; it increases linearly with time during one cycle. Thus, end expiration (EE) is approximately at phase 50%−60% (see Fig. 1). In the present work, the Advantage4D software uses phase to group series images. A recent study reported some improvement if amplitude is used instead of phase.^(28)^ We define “phase tolerance” (percent) as the width of the phase interval used to reconstruct a series at a given phase. For example, the “50%” series representing the thorax at EE could contain images from phase 47% to phase 52%. Phase tolerance would be 5% in that case.

### A.3 4D‐CT acquisition

Fig. 1 illustrates the principle of 4D‐CT acquisition. During a cine (4D‐CT) sequence, each slice of the object is exposed to X‐rays for a duration $T_{d}$ (cine duration), generally chosen to be 1 s longer than the breathing cycle (*c*) of the patient. For comparison, during a standard 3D‐CT acquisition, irradiation of a slice lasts only the time needed to collect the information enabling the reconstruction of a single image (i.e., $T_{r}$, the duration of a full rotation of the X‐ray tube). Using the information collected for a slice, an image is reconstructed every $T_{i}$ (cine interval). The number $N_{i}$ of reconstructed images for each slice position is

(1) $$N_{i}=\frac{T_{d}-T_{r}}{T_{i}}+1.$$

The images are then transferred to Advantage4D. The RPM respiratory signal file is combined with the image series: all images are sorted according to phase, and $N_{s}$ 3D‐CT series are generated, one for each reconstructed phase of the respiratory cycle. The system automatically uses proximity to the targeted phase (within a phase tolerance automatically selected and recorded) to choose the best images for reconstruction of the series.

Another important issue must be raised. The dose to the patient during a 4D‐CT examination is linearly correlated with $T_{d}$. During a conventional axial 3D‐CT examination, the delivered dose is proportional to the duration of irradiation per slice, which is usually equal to $T_{r}$. In case of 4D‐CT, this duration becomes $T_{d}$. Thus, during a 4D‐CT exam, the dose to the patient depends on the patient's breathing cycle; it is $T_{d}/T_{r}$ times higher (generally 4 or 5 times higher) than a standard axial 3D‐CT exam. We have confirmed this relationship by dose measurements not presented here. Moreover, at the end of an acquisition, the system displays a rough value of the CT dose index. By decreasing $T_{i}$, it is possible to increase the number of images reconstructed for a same $T_{d}$ and $T_{r}$ (see Equation 1) and then to reduce the phase tolerance. This approach must be balanced against these considerations:

- In the clinical routine, it is sometimes difficult to store a huge number of images (disk space considerations).
- Expanding the number of reconstructed images from the same collection of data is not necessarily relevant. Indeed, two consecutive images of the same slice will partly reflect common information, because they are built from the same CT projections. This common part, expressed as a fraction of a full rotation, is obtained from the ratio

(2) $$R=\frac{T_{r}-T_{i}}{T_{r}}.$$

As *R* approaches 1, the difference between consecutive images is smaller and may not be significant.

### B. Dynamic lung tumor phantom

We developed a dynamic lung‐tumor phantom to evaluate the performance of 4D‐CT. It is based on the use of a programmable servo motor on which is located an object equivalent to a lung tumor.

#### B.1 Programmable servo motor

A servo motor (IAI, Torrance, CA) was oriented vertically to achieve AP motion. A plastic platform was attached to the motor. The platform can carry various objects (up to 1 kg) outside of the motor plane. Thus objects can be imaged on CT without artifacts attributable to metallic parts. A computer and a programming language (SEL) can be used to implement complex 1D motions. The motion instructions are transferred from a computer to the motor through a USB link and are stored in a local memory. Motions are described as a sequence of points $\left(z_{i},\;v_{i}\right)$—that is, a position (mm) and a speed (mm/s) respectively. After reading a point $\left(z_{i},\;v_{i}\right)$, the motor goes to position $z_{i}$ at a speed equal to $v_{i}$. The system then reads another point $\left(z_{i+1},\;v_{i+1}\right)$, and so on. Sequences can be repeated as a loop to model a periodic motion. An analytic model or real patient breathing can be implemented.

#### B.2 Motion implementation

**Figure 2:**
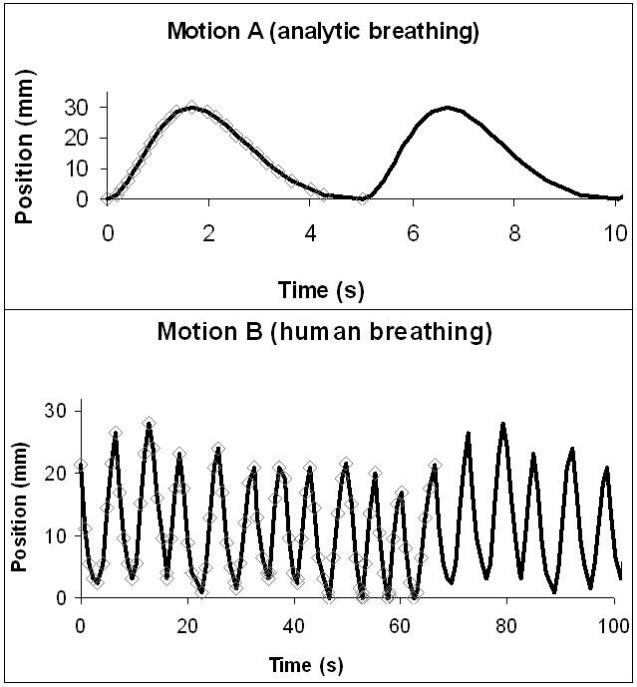
Examples of implementation of different periodic motions. The number of points (diamonds) that need to be sampled depends on the complexity of the chosen curve (solid line). Twenty‐three points can describe an analytic motion (motion A, top panel). Ninety‐six points describe a multicycle sequence (motion B, bottom panel), reproducing the real respiratory pattern of a patient, with varying peak‐to‐peak amplitudes. Motions A and B are repeated (loop) to ensure a continuous motion

Each motion to be simulated was graphically represented by plotting position as a function of time. Then, an appropriate number of points (position $z_{i}$ and speed $v_{i}$) was sampled from the theoretical curve. Because speed is constant between two points, the number of points must be large enough to describe the complexity of the shape of the curve. The number of points was increased in regions of rapid change, but also in regions with very slow motion, which are highly sensitive to speed uncertainties (see Fig. 2). In the literature, breathing motion is usually implemented using the sinusoidal model of Lujan:

(3) $$y=a\times {\text{sin}}^{n}\left(\frac{\pi }{c}t\right),$$

where *n* is a parameter equal to 1^(22)^ or to an even number^(29)^ adjusted to reflect the difference of time spent between EI and EE; *a* is the peak‐to‐peak motion amplitude (cm); the initial position at EE is 0 (except if n=1; minimum is then *‐a*); *c* is the duration of the breathing cycle or duty cycle (if n=1, then the duration is 2*c*); *y* is the position (cm); and *t* is the time. This family of models does not take into account that expiration speed is lower than inspiration speed—in other words, that breathing is asymmetric. Inspiration is known to be an active physiologic phenomenon; expiration is passive and slower.^(^ ^30^ ^,^ ^31^ ^)^ Thus, we prefer the equation

(4) $$y=a\times {\text{sin}}^{n}\begin{array}{c} {\underline{\ln(\pi +1)}}_{t}\\ \left(e\;\;\;\;\;\;\;\;\;\;\;{\;}^{c}\;\;\;\;\;\;\;-1\right), \end{array}$$

where *n* is not necessarily an integer.

**Figure 3:**
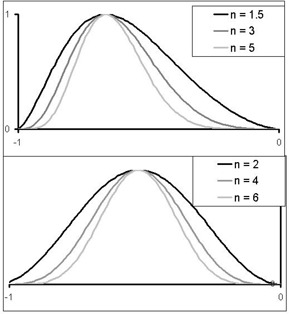
Shapes of our model for the different values of parameter *n* (top panel). The Lujan model is plotted for comparison (bottom panel). Maximum amplitude is reached at approximately x=−2/3 for our model and x=−0.5 for the Lujan model. On both panels, a=1 cm and c=1 s

In the latter model, the ratio between expiration and inspiration time (E/I) is approximately equal to 2 (see Fig. 3). This is consistent with physiologic observations. Indeed, during free breathing, E/I has been measured at 1.8.^(24)^ For a *t* varying between ‐*c* and 0, this equation describes a realistic breathing cycle. Comparison between the motion obtained from this equation and clinical data will be part of another study.

Equation 4 is not periodic, but it can be played as a loop using the servo motor. Parameters *a*, *c*, and *n* of Equation 4 were set to 3 cm, 5 s, and 2 respectively, which is consistent with a large clinical breathing cycle.^(^ ^25^ ^,^ ^26^ ^)^ One cycle of this analytic equation was sampled. Points of the curve (speed and position) were selected and implemented as a SEL sequence. Hereafter, this motion is called motion A.

Because motion A was regular and showed no variation of duty cycle or amplitude (EE or EI), we also implemented a signal from a real patient. We sampled 11 consecutive breathing cycles of a lung cancer patient, acquired with the RPM system. Hereafter, this motion is called motion B. The breathing cycle for motion B was not constant; it ranged from 5.0 s to 6.4 s. Fig. 2 illustrates the sequence of motions A and B.

Having implemented motions A and B, we placed an RPM marker on the phantom, and its motion was recorded using the RPM system. The signal was then compared with the theoretical curves (Equation 4 for motion A and the RPM signal from the patient for motion B) to check the quality of the sampling.

#### B.3 Lung tumor material

**Figure 4:**
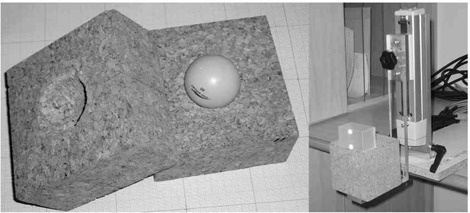
The lung tumor phantom is a ping‐pong ball (radius 20 mm) filled with resin, placed inside two half‐cubes of cork with a spherical cutout (left panel). The phantom and Real‐time Position Manager (Varian Medical Systems, Palo Alto, CA) marker are placed on the motor platform (right panel)

An equivalent to a lung tumor was placed on the motor platform (Fig. 4). It consisted of a ping‐pong ball filled with resin (radius 20 mm, density $1.02\;g•{\text{cm}}^{-3}$). The ball was placed at the centre of a cube of cork (volume 1000 cm^3^, density $0.24\;g•{\text{cm}}^{-3}$) incorporating a spherical carving (Fig. 4). Cork and resin were considered to be equivalent to lung tissue and gross tumor volume (GTV) tissue, respectively. The Hounsfield units (HUs) of the cork cube and of the ball were measured at ‐771 HUs and ‐27 HUs, with a standard deviation (SD) of 39 HUs and 28 HUs respectively. Similar phantoms had been used in previous studies.^(^ ^32^ ^,^ ^33^ ^)^

### C. Measurements

In this study, 3D‐CT is a standard 3D helical CT and 4D‐CT is an exam leading to 10 series from breathing phase 0% to phase 90%. The center of the ball was located at the CT isocenter. This position was defined as the rest position (no motion) and the origin of the AP motion axis (EE). For all acquisitions, $T_{r}$ was set to 1 s, and the slice width was set to 5 mm to be consistent with our clinical practice. The CT charge was set to 100 mA, and the voltage to 120 kV. Several 3D‐CT and 4D‐CT series were captured for various combinations of $T_{d}$ and $T_{i}$ values, without and with motion of the phantom (motion A or motion B). On all series, the volume and AP limits of the ball were studied. The study was restricted to AP motion.

#### C.1 Volume and AP limits

On each series (3D‐CT and 4D‐CT acquisitions), the ball was automatically segmented using the thresholding tool on an Advantage Windows workstation (GE Healthcare). To extract the ball from the cork, threshold values were set to arbitrary values (from −503 HU to +779 HU). Volume was then computed automatically and compared to the geometric volume of the ball (33.51 cm^3^). This operation was first performed on 3D‐CT with motion A, motion B, and without any motion (three series each). Mean values and SDs were computed. Then, the ball volume was also computed for the 10 phases of a 4D‐CT series using motion A. Three values of $T_{d}$ were used: 4, 6, and 10 s ($T_{i}=0.5\;s$ and $T_{r}=1\;s$). Positions of the AP limits of the ball were also recorded. One 3D‐CT and two 4D‐CT series ($T_{i}=0.2\;s,\;T_{r}=1\;s,\;T_{d}=6\;s$ and 3 s) were acquired during motion A. A series with a small value of $T_{d}(3\;s)$ was captured to see the behavior of 4D‐CT when $T_{d}$ is smaller than the breathing cycle.

Using a lung visualization window and level (W/L=−600/1600), the anterior and posterior limits of the ball were manually measured on the workstation. These AP limits, expressed in the coordinate system of the CT room, were compared to the theoretical position extracted from the programmed SEL sequence. The ball diameter was derived from the AP limits and also compared to the real value (40 mm). This measurement was used as an assessment of both accuracy and precision because the *real* position of the ball was known for all phases of all acquisitions.

#### C.2 Merged series

Finally, to evaluate the possibility of contouring a consistent ITV using 4D‐CT for a gated or non‐gated treatment, we studied volumes on series created by merging images belonging to different phases. On a 4D‐CT $\left(T_{d}=6\;s,\;T_{i}=0.5\;s,\;T_{r}=1\;s\right)$ merging was achieved by using two different tools of Advantage4D (v1.4): maximum intensity projection (MIP) and average projection (AVE). Series resulting from merging phases 40%+50%+60% and 30%+40%+50%+60%+70% were called small window at end expiration (SWEE) and large window at end expiration (LWEE) respectively. Other series were created at EI: small window at end inspiration (SWEI: 90%+0%+10%) and large window at end inspiration (LWEI: 80%+90%+0%+10%+20%). These series allowed a gated treatment with two widths of gating window (20% and 40%) centered at the extreme phases (EE and EI) of the breathing cycle to be modeled. A series created by merging all series (0% to 90%) was also constructed to model a non‐gated treatment.

**Figure 5:**
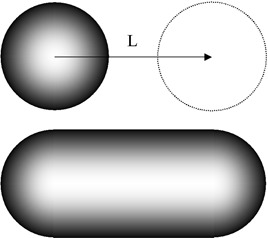
Representation of the theoretical volume of a moving sphere. If the excursion of the sphere is *L* (top panel), then Equation 5 gives the volume of the resulting shape (bottom panel)

On the merged series, the resulting shape of the ball was automatically segmented. Then the volume of this “apparent ball” was computed using the Advantage Windows workstation and was compared to a theoretical ITV $\left({\text{ITV}}_{\text{th}}\right)$ computed using the equation

(5) $$ITV_{th}=\frac{4}{3}\pi r^{3}+L\times \pi r^{2},$$

where *r* is the radius of the ball and *L* is the ball excursion within the corresponding gating window. This simple formula gave the volume of a shape composed of a sphere plus a cylinder with the same radius (see Fig. 5). We deduced *L* from the programmed sequence.

## III. RESULTS

### A. Quality of the sampled motion

**Figure 6:**
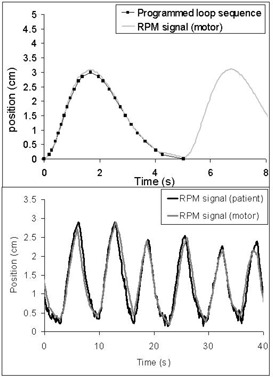
Comparison of the programmed motion A (sequence of points) and the corresponding acquired Real‐time Position Manager (RPM: Varian Medical Systems, Palo Alto, CA) signal using the motor (top panel). For motion B, the RPM signal of the motor is compared to the RPM source acquired with a patient (bottom panel)

Fig. 6 shows the motions (A and B) produced by the motor and acquired with the RPM system. Motion A and motion B were compared to the expected signals (i.e., analytic programmed curve and RPM signal of a real patient respectively). Time scales were matched using a particular point (i.e., the first EI).

Motion A was well reproduced; the discrepancy between the RPM file and the programmed sequence was less than 1 mm. For motion B, the sampling rate used to program the sequence was not high enough to reproduce quick variations observed for the patient at EE. And some of the variation was attributable to couch movement causing vibration in the RPM camera, which was attached to the CT couch. Thus, we assumed that motion B modeled a realistic chest motion.

### B. Volume

Table 1 reports the volume of the ball measured on three repeated standard 3D‐CT series with and without motion. Mean values of the volume were slightly larger in presence of motion and the SDs were also larger. Mean values were $34.1\pm 0.1\;{\text{cm}}^{3},\;35.8\pm 1.6\;{\text{cm}}^{3}$, and $34.9\pm 0.7\;{\text{cm}}^{3}$ for the phantom at rest, animated with motion A, and animated with motion B respectively.

**Table 1:** Volume of the ball on three consecutive three‐dimensional computed tomography acquisitions; error is defined as the relative difference in the acquisitions as compared with the geometric volume of the ball (33.5 cm^3^)

	No motion	Motion A	Motion B
Ball volume (cm^3^)			
Acquisition 1	34.2	34.0	34.1
Acquisition 2	34.1	36.2	35.3
Acquisition 3	34.0	37.2	35.3
Mean±SD	34.1±0.1	35.7±1.6	34.8±0.7
Error on volume (%)	1.8	6.7	4.1

**Figure 7:**
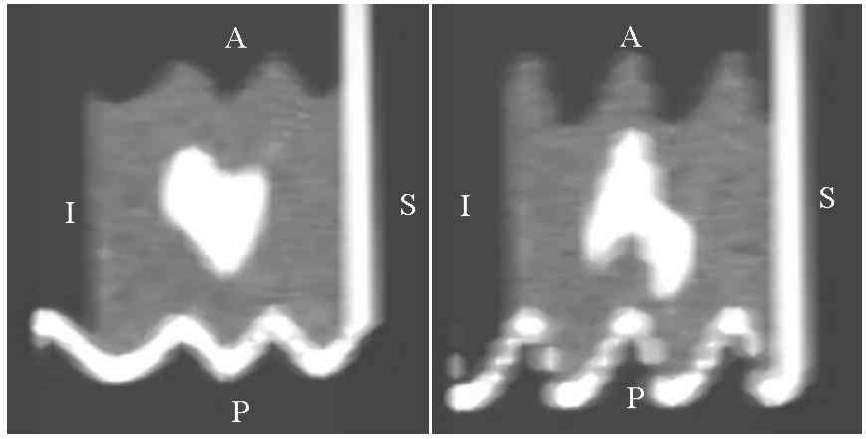
Sagittal reconstruction of the phantom using helical three‐dimensional computed tomography for motions A (left panel) and B (right panel). Image quality is poor and the shapes of the motion curves can clearly be seen on the shape of the horizontal platform

It should be noted that the volume error is not correlated with the reduction in image quality induced by AP motion. Fig. 7 shows sagittal reconstructions of helical 3D‐CT of the phantom during motions A and B. Images were strongly degraded by the movement, but the error on volume measurement was only 10.9% and 4.1% for motion A and motion B respectively.

**Figure 8:**
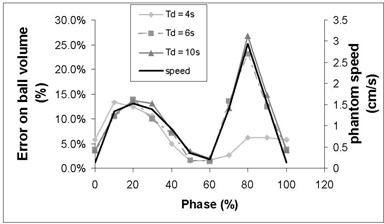
Error on ball volume assessment using four‐dimensional computed tomography during motion A (comparison is to the real volume). For all series, $T_{r}=1\;s\;\text{and}\;T_{i}=0.5\;s$. The corresponding speed of the phantom is also plotted

**Figure 9:**
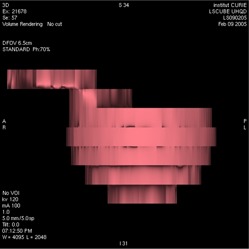
Volume rendering of the ball on four‐dimensional computed tomography at phase 70% (motion A). $T_{r}=1\;s,\;T_{i}=0.5\;s,\;T_{d}=4\;s$. The $T_{d}$ value is too small (less than the breathing cycle duration, *c*), and large inconsistencies between the slices are observed

When using 4D‐CT, the volume of the ball reconstruction depended strongly on the phase. Fig. 8 shows the error in the ball volume measured for various phases of several 4D‐CT examinations. When $T_{d}$ is large enough, a clear correlation appears between the absolute value of the speed of the object (plotted on the same figure) and the apparent volume. Speed was computed using the SEL sequence (average on the corresponding 10% phase interval). At mid‐inspiration (phase 80%), speed and error on volume assessment were at maximum. For a small value of $T_{d}$ (4 s), the error on the volume was smaller. However, the smaller error did not indicate better image quality. Actually, as Fig. 9 illustrates, large phase tolerance led to important inconsistency of reconstructed ball position from slice to slice.

### C. AP limits

**Figure 10:**
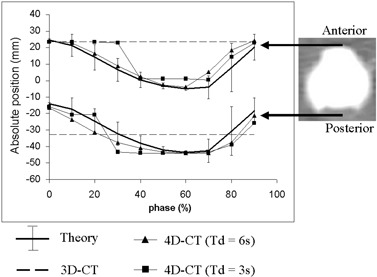
Anterior (top) and posterior (bottom) limits of the ball during motion A. Thick solid lines represent the programmed sequence. The vertical error bars represent the distance covered by the ball during 1 s for each phase. The dashed lines represent the limits of the ball on standard helical three‐dimensional computed tomography (3D‐CT—non‐variant with the phase). Two four‐dimensional computed tomography (4D‐CT) exams are plotted for $T_{i}=0.2s$ and $T_{d}$ equal to 3 s (squares) and 6 s (triangles)

Fig. 10 illustrates the anterior and posterior limits of the ball during motion A for various CT acquisitions (3D‐ and 4D‐CT) in the CT coordinate system (origin was the isocenter). The theoretical motion—that is, the motion based on the programmed position of the ball during motion A—was also plotted. Errors on the position assessment of two equivalent points (anterior and posterior limits of the ball) were very different and thus were not consistent. Consequently, the diameter of the ball was strongly overestimated on 3D‐CT (+41% as compared with the real diameter). This result depends on the time of the beginning of the CT acquisition; different results could be obtained for other successive acquisitions.

Acquisitions by 4D‐CT gave a more consistent position of the ball during its motion. For a chosen set of parameters $\left(T_{d}=6\;s\right)$, the error in the position of the anterior and posterior limits ranged from 0.4 mm to 10.6 mm for all phases, but only from 0.4 mm to 2.6 mm for the extreme phases of the breathing cycle (phases 0% and 60%) generally chosen for treatment. The diameter of the ball was overestimated by 0%−41% during the breathing cycle, but this overestimation was smaller than 2% for the two extreme phases. On Fig. 10, the vertical error bars plotted on the theoretical curve represent the distance covered by the ball in 1 s (duration of one image acquisition). When $T_{d}$ was 6 s, the apparent position of the ball on 4D‐CT was within these limits in almost all cases. When $T_{d}$ was too small (3 s, squares on the figure), the reconstructed position was no longer within the error bars.

### D. Merging series

**Figure 11:**
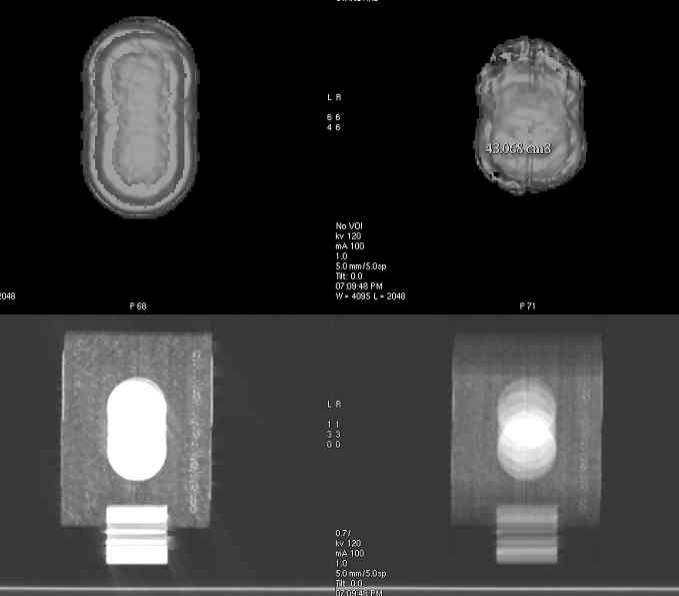
Volume rendering of the ball (top panel) and central axial slice of the phantom (bottom panel) during motion A. Images are obtained by merging all the phases of a four‐dimensional computed tomography (4D‐CT) exam (10 phases) using two different algorithms: maximum intensity projection (MIP, left) and average projection (AVE, right). Acquisition parameters were $T_{d}=6\;s,\;T_{r}=1\;s$, and $T_{i}=0.5\;s$

**Figure 12:**
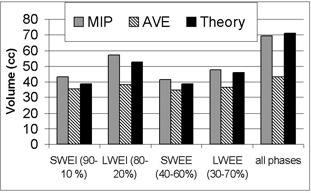
Apparent volume of the ball computed from merged series of four‐dimensional computed tomography. The merging is performed using various phase combinations and various algorithms. The “all phases” series corresponds to the merging of all 10 phases. The expected value from Equation 5 (“theory”) is also plotted for comparison. MIP is maximum intensity projection, AVE is average projection, SWEI is small window centered at end inspiration, LWEI is large window centered at end‐inspiration, SWEE is small window centered at end‐expiration, and LWEE is large window centered at end‐expiration

Several series from 4D‐CT were merged to create new series. The AVE and MIP algorithms were used. Fig. 11 shows, as an example, the result of merging all phases (0% to 90%) of a 4D‐CT of the moving phantom (motion A). Fig. 12 shows the apparent volume of the ball extracted from merged series of 4D‐CT. The “all phases” series corresponds to the merging of all 10 phases illustrated on Fig. 11. Compared to the theoretical ${\text{ITV}}_{\text{th}}$ for all the series, the error on volume assessment ranged from −40% to −9% for the AVE algorithm and from −3% to 12% for the MIP algorithm.

## IV. DISCUSSION

We developed a tool that permits various tests for quality control in 4D radiotherapy. Several objects can be placed on the platform. Motion is linear and can be achieved in the AP or SI direction, or along an oblique direction. Although the possibility of simulating 3D trajectories could be considered more general and representative of the clinical reality, we decided to investigate only linear motion, to facilitate the quality assessment. A 3D motion of our phantom would have the consequence of mixing various types of artifacts, compromising the conclusions.

Rietzel et al. used a similar approach to extensively study the case of SI motion.^(22)^ In the presence of SI motion, the main source of artifacts is movement of a point of an object from one slice to the next. If the SI motion is large, the typical artifact is the separation of small objects into independent structures or a spiral‐shaped blurring on axial images.^(23)^

The present work focused on artifacts observed on CT images in presence of AP motion. For a complex 3D motion, the resulting artifacts would be a combination of SI‐specific and AP‐specific artifacts. For motion along the AP direction, the CT image can be affected in two ways: blurring because of object motion during a tube rotation at a given table position (intraslice artifact), and AP position error from slice to slice (interslice artifact), illustrated in Fig. 9, for example. Notice that, for our four‐slice CT scanner, four consecutive slices have the same phase, and no interslice artifacts appear for these four slices. Intraslice and interslice artifacts are discussed in the next two paragraphs. In the discussion, artifacts are to be understood as problems either in the accurate assessment of the absolute position of a point or in the quality of the shape rendering of an object.

Intraslice artifacts depend on the speed of the object during acquisition. These artifacts deteriorate the quality of the object's image by increasing its apparent size and decreasing the HUs of a portion of it. These artifacts are of the same nature as the partial volume effect, well known by radiologists. In that case, the volume obtained by automatic segmentation becomes highly sensitive to the chosen thresholds.^(23)^ These artifacts can be reduced by increasing the speed of source rotation. From this point of view, $T_{r}$ should be set at its minimum value. Compared to an axial 3D‐CT, 4D‐CT does not reduce intraslice artifacts, because the duration used to acquire one image $\left(T_{r}\right)$ is the same for both sequences. However, 4D‐CT allows for sorting and selection of images at the phases when the effect is minimized by relative immobility: EE or EI. Apparent volume of the object is a pertinent indicator of intraslice artifacts. Fig. 8 shows clearly that the volume error is strongly correlated with the speed of the object. This error is minimum for EI (phase 0%) and EE (phases 50% and 60%). The volume of the same ball measured from the 3D‐CT series is not overestimated (Table 1) even if the image quality is poor (Fig. 7). Indeed, 3D‐CT images are more degraded by interslice artifacts, and the benefits of 4D‐CT are linked to this second source of image degradation.

Interslice artifacts occur because the various images of a CT series are not acquired at the same phase of the breathing cycle. For a 3D‐CT, object reconstruction is performed from images acquired at randomly selected phases, as illustrated in Fig. 7. If a small phase tolerance is used, 4D‐CT allows objects to be reconstructed for individual phases. Knowing the breathing cycle duration, *c*, $T_{r}$, $T_{d}$, and $T_{i}$ have to be properly set to avoid a lack of data that leads to large object distortions (Fig. 9). Volume is not a good indicator for these interslice artifacts. Overestimation of ball volume was just 2.6% in Fig. 9 and 10.9% and 5.4% in the images of Fig. 7. In the present work, the absolute position of a moving object in a CT series was investigated. Fig. 10 shows that interslice artifacts can be reduced by using 4D‐CT. The value of $T_{d}$ should be large enough to ensure complete coverage of the entire breathing cycle. Generally $T_{d}$ is chosen as $\left(c+T_{r}\right)$.

Interslice artifacts can also be observed in cases of irregular breathing—that is, when breathing cycles have different amplitudes (e.g., as in motion B). Two images could then have the same phase value even if the position of the object was different during the acquisition. This last source of error can be controlled by plotting phase against amplitude of the respiratory signal as proposed by Rietzel et al.^(22)^ Interslice artifacts can also be minimized if patient breathing is more reproducible. Reproducibility can be achieved by using audio and video instructions^(34)^ or by training the patient in one or more sessions,^(35)^ or both.

When the influence of SI motion is analyzed, slice thickness should be considered. In the present study, we used a 5‐mm slice thickness. This thickness could be reduced to improve the SI resolution, but a reduction would have no effect on the artifacts induced by AP motion.

Merging several CT series at various phases seems a pertinent way to obtain the volume and the shape of the trajectory of an object in motion. This approach can be helpful in planning a treatment with or without gated irradiation. In absence of gated treatment, all phases can be merged to create an ITV from the combination of the 10 GTVs. Such a solution is being used in clinical cases.^(36)^

More recently, Leter et al. showed that personalized ITVs permit more significant sparing of OARs, as compared with ITVs created using an empiric motion margin^(37)^ (study based on 10 patients). For this purpose, the MIP algorithm gives a better evaluation of volume and shape than the AVE algorithm does (Figs. 11 and 12). Because MIP overestimates HU values of the media, the AVE algorithm could be used to create a series for dose computation. In the case of gated treatment, we currently use the MIP algorithm to create an ITV corresponding to the residual motion within the gating window. Generally, we merge series at phases 90%, 0%, and 10% (SWEI) to create a plan that will be used for a treatment with a gating window of about 20%−30% centered on the patient's EI. It must be noted that this definition of ITV is not consistent with the recommendations of the International Commission on Radiation Units and Measurements,^(^ ^38^ ^–^ ^40^ ^)^ because it does not take into account the microscopic extensions of the visible GTV. Whether the margin for microscopic extension should be added before or after taking into account the internal movement of the GTV is open to discussion.

## V. CONCLUSION

Various 4D‐CT acquisition parameters were discussed, and the following rules were validated:

- $T_{r}$, the duration of a tube rotation, should be set as small as possible, which may necessitate higher mA settings to keep a large signal‐to‐noise ratio.
- The cine duration $\left(T_{i}\right)$ should be set at $c+T_{r}$.

Under these conditions, the AP accuracy of 4D‐CT (assessment of the absolute position of a point) was found to range from 0.4 mm to 2.6 mm for extreme phases of the breathing cycle (phases 0% and 60%). Using these guidelines, geometric accuracy will be better for clinical situations in which the motion amplitude is smaller. Particular attention should be paid to the value of $T_{d}$, which determines the dose to the patient. The cine interval, $T_{r}$, can be chosen to be as small as possible. But a small interval increases the number of images to be stored. In the end, using 4D‐CT, we propose a methodology that consists of merging GTV images to create an ITV for gated or non‐gated treatment. For this purpose, the MIP algorithm should be preferred over the AVE algorithm, because MIP gave a more realistic evaluation of the merged volume in presence of motion.

## ACKNOWLEDGMENTS

Our work was supported in part by grant STIC 2003 from the French Minister of Health and from La Ligue contre le Cancer–Comité de Paris. The authors thank J.P. Lacomme, J.Y. Kristner, A. Rousseau, and T. Lemoine for technical support and Eike Rietzel for his contribution to the design of Fig. 1.
